# The prognostic value of interleukin-17 in lung cancer: A systematic review with meta-analysis based on Chinese patients

**DOI:** 10.1371/journal.pone.0185168

**Published:** 2017-09-21

**Authors:** Xiao-fei Wang, Yi-tong Zhu, Jia-jia Wang, Da-xiong Zeng, Chuan-yong Mu, Yan-bin Chen, Wei Lei, Ye-han Zhu, Jian-an Huang

**Affiliations:** 1 Department of Respiratory Medicine, the First Affiliated Hospital of Soochow University, Suzhou, Jiangsu, China; 2 Department of Boxi Medical Center, the First Affiliated Hospital of Soochow University, Suzhou, Jiangsu, China; Sapporo Ika Daigaku, JAPAN

## Abstract

**Background:**

Interleukin-17 (IL-17) plays an important role in cancer progression. Previous studies remained controversial regarding the correlation between IL-17 expression and lung cancer (LC) prognosis. To comprehensively and quantitatively summarize the prognostic value of IL-17 expression in LC patients, a systematic review and meta-analysis were performed.

**Methods:**

We identified the relevant literatures by searching the PubMed, EMBASE, Cochrane Library, SinoMed, China National Knowledge Infrastructure (CNKI) and Wanfang Data databases, up until April 1, 2017. Overall survival (OS), disease free survival (DFS) and clinicopathological characteristics were collected from relevant studies. Pooled hazard ratios (HR) and corresponding 95% confidence intervals (CI) were calculated to estimate the effective value of IL-17 expression on clinical outcomes.

**Results:**

Six studies containing 479 Chinese LC patients were involved in this meta-analysis. The results indicated high IL-17 expression was independently correlated with poorer OS (HR = 1.82, 95% CI 1.44–2.29, P < 0.00001) and shorter DFS (HR = 2.41, 95% CI 1.42–4.08, P = 0.001) in LC patients. Further, when stratified by LC histological type (non-small cell lung cancer and small cell lung cancer), tumor stage (Ⅰ-Ⅲ,Ⅰ-Ⅳ and Ⅳ), detection specimen (serum, intratumoral tissue and pleural effusion), test method (immunological histological chemistry and enzyme linked immunosorbent assay), and HR estimated method (reported and estimated), all of the results were statistically significant. These data indicated that elevated IL-17 expression is correlated with poor clinical outcomes in LC. The meta-analysis did not show heterogeneity or publication bias.

**Conclusions:**

The present meta-analysis revealed that high IL-17 expression was an indicator of poor prognosis for Chinese patients with LC. It could potentially help to assess patients’ prognosis and estimate treatment efficacy in therapeutic interventions.

## Introduction

Lung cancer (LC) has become a major public health problem worldwide, and there are about 1.8 million newly diagnosed LC patients every year [1]. LC can be divided into two types: small cell lung cancer (SCLC) and non-small lung cancer (NSCLC). It is the leading cause of cancer-related death in recent decades [2]. With an overall five-year survival rate of approximately 17%, the prognosis for LC patients is generally poor, and remained unchanged these days [2,3]. In China, LC incidence rate has increased by 51.8% in 2015 due to the prevalence of smoking and growingly serious environmental pollution than 2005 [4,5]. Although many novel treatment approaches have been developed for LC, there are still no significant improvements in patients’ outcomes [6,7]. Furthermore, not all patients can benefit from standard chemotherapy or new targeted therapies. Therefore, finding of efficient biomarkers to choose suitable treatments for individual patient is very significant for patients to acquire the maximum benefit of therapies [8,9].

Interleukin-17 (IL-17) is a pro-inflammatory cytokine group of ligands which is mainly secreted by activated CD4(+) T-helper cells known as Th17 cells, macrophages and CD8(+) T cells [10]. IL-17 has several biologic functions, involving the induction of IL-6, IL-8, IL-18, TNF-α and the stimulation of vascular endothelial cell migration and neoangiogenesis [11,12]. IL-17 plays an essential role in cancer progression. On one side, IL-17 could promote tumor progression by antiapoptosis and angiogenesis [13–15]. On the other side, it could advance effector cytotoxic T lymphocytes generation and enhance anti-tumor immune responses [16–18]. Elevated serum levels of IL-17 are relevant with disease severity, deteriorating overall survival (OS) in several kinds of cancers. Given the significance impact of IL-17 on the development and progression of many types of cancer, a growing body of evidence has revealed the prognostic role of IL-17 levels in patients with LC [19–24]. However, the published studies were controversial and the prognostic role of IL-17 in LC remained still unknown. To evaluate the relationship between IL-17 and its prognosis value in patients with LC, a systematic meta-analysis of the current published research was performed.

## Materials and methods

### Search strategy and case retrieval

We researched related articles in the PubMed, EMBASE, Cochrane Library, SinoMed, China National Knowledge Infrastructure (CNKI) and Wanfang Data databases and investigated the correlation between IL-17 expression and survival in LC patients. During the case retrieval, no language restriction was imposed. Articles were searched by using the following MeSH terms or keywords: “lung cancer”, “lung neoplasm”, “Interleukin-17”, “IL-17” and “prognosis” separately and in combination. Review studies and reference lists relevant to those articles were also reviewed. Study cases eligible for inclusion in this meta-analysis share the following criteria: (1) patients with cytologically or histologically confirmed diagnosis of LC, (2) measured IL-17 protein expression, (3) assessed the correlation of IL-17 with survival outcome in LC, (4) enrolled more than 30 patients, (5) provided sufficient data to calculate hazard ratios (HR) and 95% confidence intervals (95% CI) according to IL-17 expression, (6) in overlapped research published by the same author, we chose the most complete and the newest research. Two researchers (WXF, ZYT) independently assessed the titles and abstracts, and excluded the irrelevant research; then the full-texts were scrutinized by the review team. And the studies were selected for meta-analysis according to the pre-specified criteria. Two reviewers (WJJ, ZDX) determined study eligibility independently. Disagreements were resolved by consensus.

### Data extraction and quality assessment

For statistical analysis, the following items were extracted from each study case: name of first author and year of publication; study population characteristics such as number of patients, country, age, gender and follow-up time; tumor data such as histologic type and TNM stage; variables such as test method, detection specimen, cut-off value for the IL-17 expression; prognostic data such as HR and 95% CI for prognosis. Items that could not be obtained were described as “not reported (NR)”.

Two reviewers (WXF, ZYT) independently graded the methodological quality of the selected studies by the Newcastle-Ottawa Scale (NOS) criteria to ensure consistency in reviewing and reporting results. The studies were assessed three broad perspectives on the following: (1) the selection of the study groups; (2) the comparability of the groups; (3) the ascertainment of either the exposure or outcome of interest. A study was graded as low, moderate or high quality with the score 0 ~ 3, 4 ~ 6 and 7 ~ 9, respectively. Disagreements during quality assessment will be settled by discussion, or a third investigator was consulted.

### Statistical analysis

The level of correlation between IL-17 expression and prognosis can be indicated by HR. By convention, a combined HR > 1.00 suggested an adverse prognosis in patients with increased expression of IL-17, and the effect of IL-17 expression on prognosis was supposed to be statistically significant when the 95% CI for the overall HR did not overlap 1. To estimate the prognostic role of IL-17 expression, the overall HR and 95% CI were evaluated for higher IL-17 expression. The pooled HR was initially demonstrated using forest plots graphically. Subgroup analysis was then performed when the risk (HR) was significant (P < 0.05). We first extracted HR and 95% CI that could be directly obtained from the original article by using survival analysis. For those studies which didn’t report HR and 95% CI, an estimated value was generated indirectly from other supplied data by the methods described by Tierney et al [25]. If the data was only available in the form of graphics, we digitized Kaplan-Meier curves from the articles (GetData Graph Digitizer 2.24 http://getdata-graph-digitizer.com) and extracted relative data to calculate HR and 95% CI using previously described methods[26,25]. If a study described both univariate and multivariate analysis, we chose the latter because survival clinical outcomes in LC were influenced by confounding factors.

The extracted data across the studies was analyzed using the Cochran’s Q test and Higgins I^2^ statistic. If study heterogeneity was known to be absent, we applied a fixed effects model. If significant heterogeneity (P < 0.05 or I^2^ test exhibited > 50%) was considered present and we performed the random effects model instead [27].

Publication bias was examined using a funnel plot [28]. To evaluate the stability of the results, a sensitivity analysis was employed, in which one research at a time was removed to check its individual impact on the combined HR. All p-values were 2-sided; p < 0.05 was considered statistically significant.

Subgroup analysis was conducted to evaluate the significance of IL-17 expression as a prognostic indicator for LC patients in studies of histological type, disease stage, test method, detection specimen for IL-17 expression and the method used to obtain the HR. We also performed tests of interaction to detect differences between subgroups.

These analyses were conducted with Review Manager Version 5.3 software (http://ims.cochrane.org/revm-an).

## Results

### Study selection and characteristics

**Fig 1:**
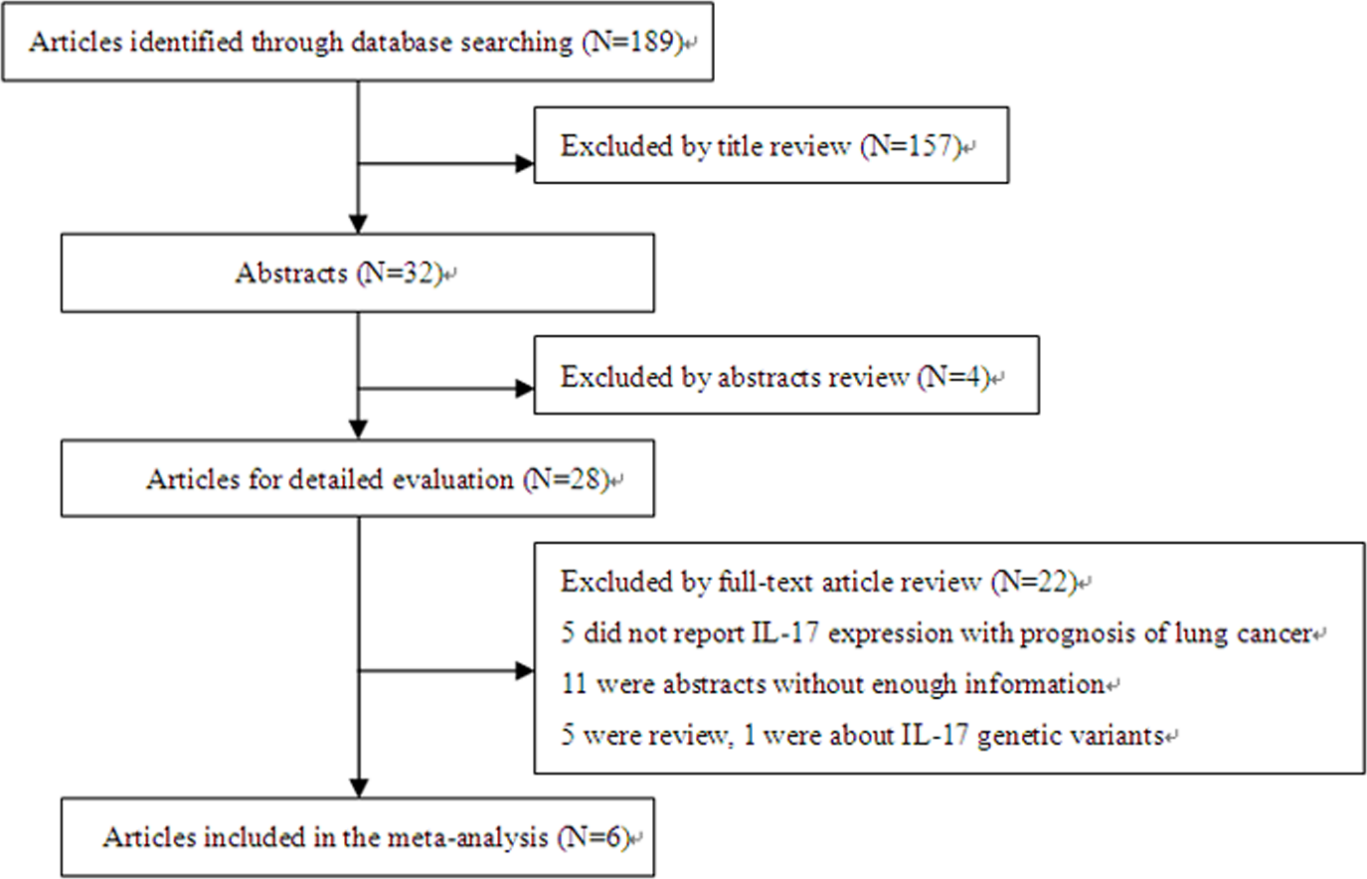
Flow chart of article selection for systematic review and meta-analysis.

A total of 189 articles were selected after initial databases search. The titles and abstracts of relevant literatures were separately reviewed by two authors. Finally, 28 out of 189 study cases were sent for full-text assessment. Six study cases with sufficient data or with information that could be calculated indirectly were evaluated in the current meta-analysis [19–24]. The flow diagram of the literature selection procedure is described in Fig 1, and the baseline characteristics of the evaluable articles are summarized in Table 1. In the meta-analysis, data from 479 patients with LC was included, which was extracted from six selected study cases published between 2010 and 2015. The sample size of these studies varied from 43 to 128 patients. The age of the patients ranged from 29 to 77, and the overall proportion of males was 62.42%. The six selected study cases were all from China. In all the studies, patients did not receive adjuvant therapy prior to surgery, and the specimens were collected before any treatment. Among these, four studies involved cases with the histopathology of NSCLC, one case studied SCLC, and the other one included both NSCLC and SCLC. Three studies reported all stages of the disease (Ⅰ-Ⅳ), two studies included stages of Ⅰ to Ⅲ and one study involved only advanced stage of the disease (Ⅳ(M1a/M1b)). Two studies assessed IL-17 by immunological histological chemistry (IHC) [19,20] and four studies investigated IL-17 using enzyme linked immunosorbent assay (ELISA) [21–24]. In addition, two studies detected IL-17 expression using LC tissues [19,20], two studies investigated IL-17 level by serums [21,23,24] and the remaining one identified it with pleural effusions [22]. OS was acquired in six studies [19–24] and disease free survival (DFS) was obtained in two studies [19,23]. Five cases provided HR and 95% CI values directly from multivariate Cox analysis, while HR were estimated from Kaplan–Meier survival curves in the last case.

**Table 1:** Main characteristics and results of the eligible studies.

Author	Year	Histological type	NO. (male/female)	Age (year)	Tumor stage	Assessment IL-17	Test method	Cut-off (IL-17 high)	High expression (%)	Median follow-up(months)	Prognostic condition	HR Estimation	OS/DFS HR (95% CI)
**Chen X [19]**	2010	NSCLC	52(41/11)	Median51.9	Ⅰ-Ⅱ33/Ⅲ19	Intratumoral cells	IHC	Positive cells>5%	25 (48.1%)	Not reported	OS/DFS	Reported	OS2.335(1.017–5.36) /DFS2.345(1.012–5.435)
**Zhang GQ[20]**	2010	NSCLC	102/(66/36)	Mean65	Ⅰ42/Ⅱ27/Ⅲ33	Intratumoral cells	IHC	Density of positive cells	72 (70.5%)	30.2	OS	Estimation	OS 1.387(1.013–1.828)
**Xu CH[21]**	2014	NSCLC	128/(77/51)	Median54	Ⅰ-Ⅱ85/Ⅲ-Ⅳ43	Serum	ELISA	≥16 pg/ml	90 (70.3%)	24	OS	Reported	OS2.335(1.017–5.36)
**Xu CH[22]**	2014	NSCLC and SCLC	78(36/42)	Median65	M1a54/M1b24	Pleural effusion	ELISA	>15pg/ml	Not reported	Not reported	OS	Reported	OS3.040(1.348–6.849)
**Pan B[23]**	2015	NSCLC	43/(27/16)	≥65 29 67%	Ⅰ-Ⅱ24/Ⅲ6/Ⅳ13	Serum	ELISA	>15pg/ml	Not reported	Not reported	OS/DFS	Reported	OS2.019(1.009–4.042)/DFS2.447(1.244–4.814)
**Lin QY[24]**	2015	SCLC	76/(52/24)	Median51	Ⅰ-Ⅱ27/Ⅲ-Ⅳ49	Serum	ELISA	>24.93pg/ml	Not reported	Not reported	OS	Reported	OS3.056(1.49–6.269)

Note: The six selected study cases were all from China. Abbreviation: No.: Patients number; NSCLC: non-small cell lung cancer; SCLC: small cell lung cancer; IHC: immunological histological chemistry; ELISA: enzyme linked immunosorbent assay; IL-17: interleukin-17; OS: overall survival; DFS: disease free survival; HR: hazard ratio; CI: confidence interval.

### Qualitative assessment

The NOS scores of the included studies ranged from 7 to 9 and the methodological quality of the 6 eligible studies were summarized in Table 2.

**Table 2:** Study quality assessment based on the NEWCASTLE-OTTAWA QUALITY ASSESSMENT SCALE (NOS).

Study	Year	Selection	Comparability	Outcome	Total scores
**Chen X [19]**	2010	4	3	1	8
**Zhang GQ [20]**	2012	3	1	3	7
**Xu CH [21]**	2014	4	3	1	8
**Xu CH [22]**	2014	4	3	1	8
**Pan B [23]**	2015	4	3	2	9
**Lin QY [24]**	2015	4	3	1	8

### Meta-analysis

**Fig 2:**
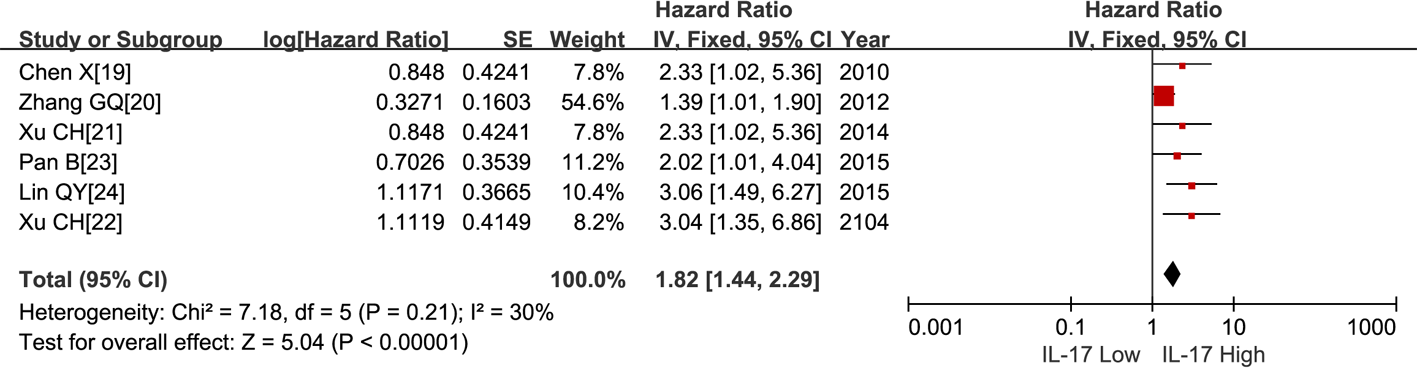
Meta-analysis comparing IL-17 expression and overall survival (OS) in lung cancer patients. The individual and pooled HR with 95% CI was shown by forest plot. HR > 1 implied worse OS for the group with high IL-17 expression.

**Fig 3:**
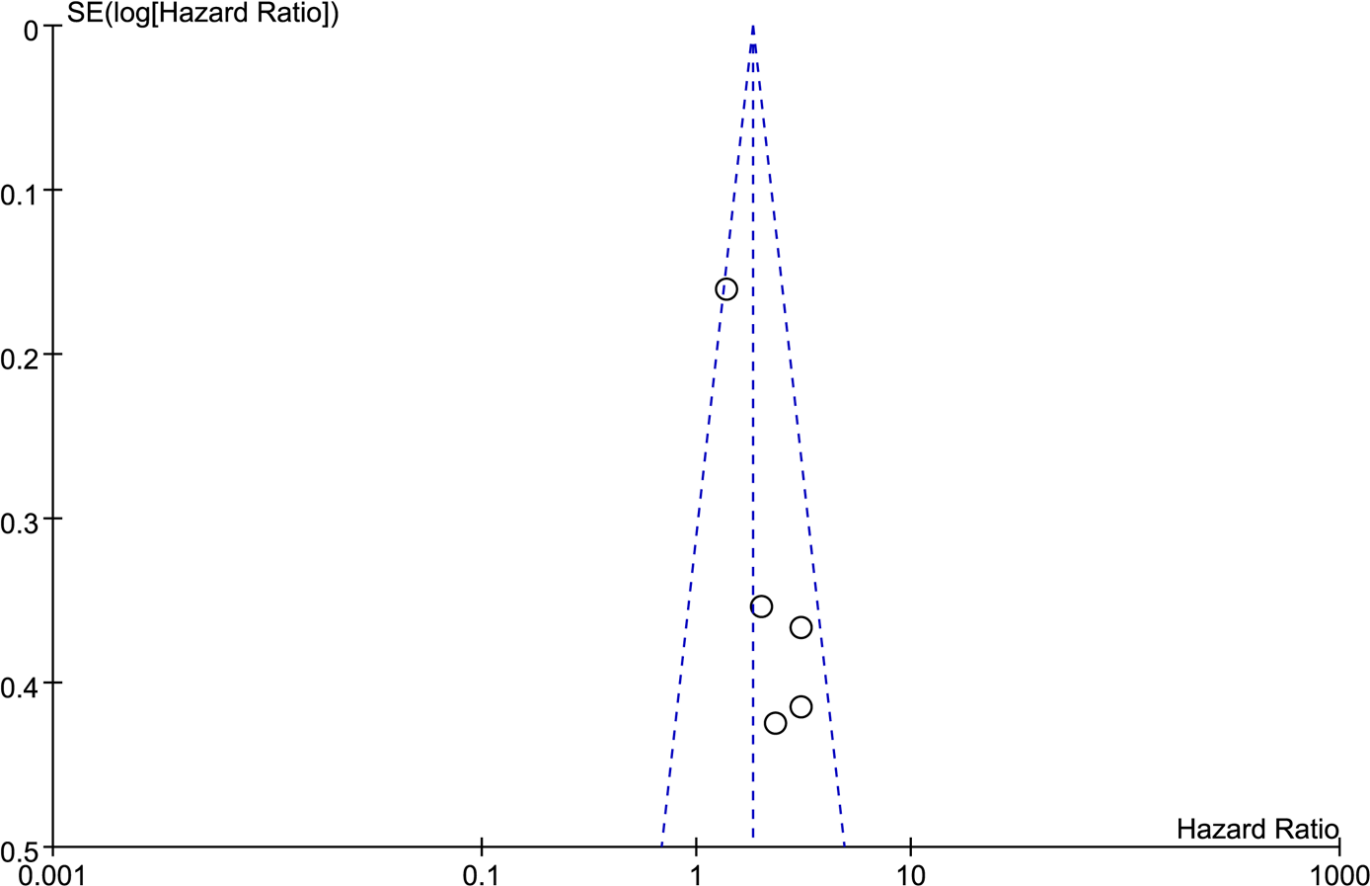
Funnel plot was used to visualize a potential publication bias on overall survival (OS).

**Fig 4:**
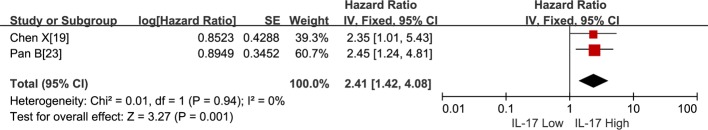
Meta-analysis comparing IL-17 expression and disease free survival (DFS) in lung cancer patients. The individual and pooled HR with 95% CI was shown by forest plot. HR > 1 implied worse DFS for the group with high IL-17 expression.

**Fig 5:**
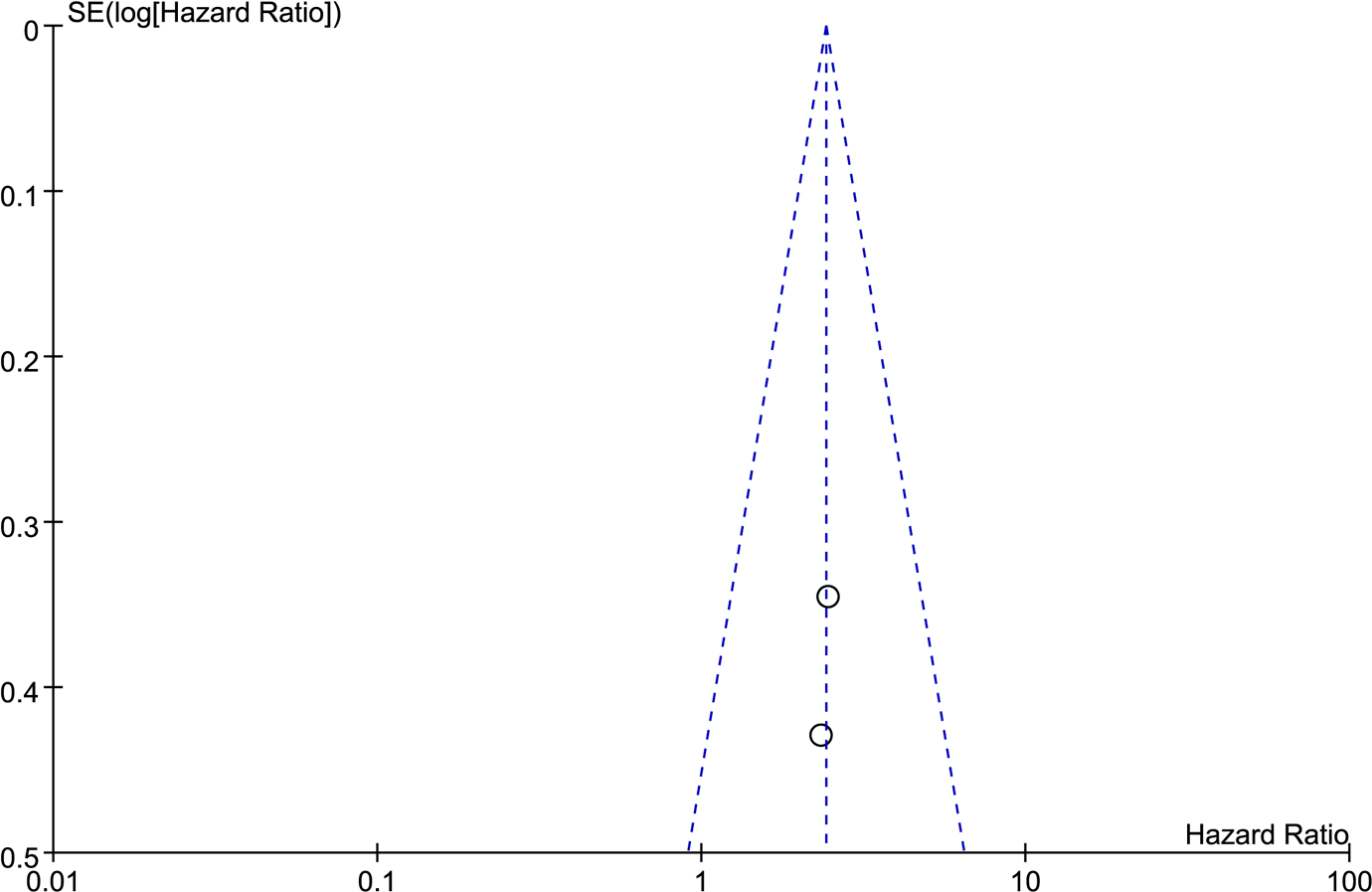
Funnel plot was used to visualize a potential publication bias on disease free survival (DFS).

**Fig 6:**
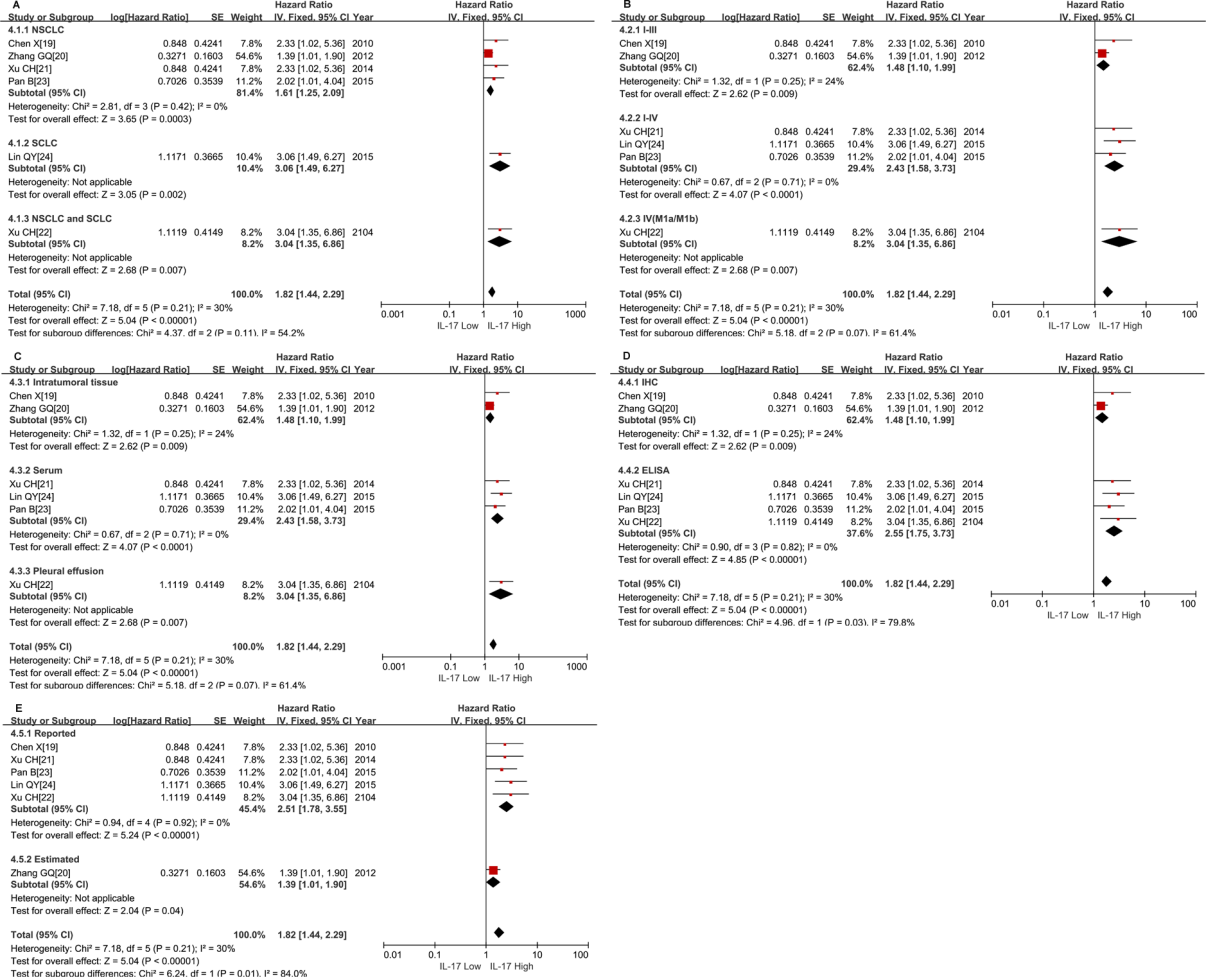
**Meta-analysis between IL-17 overexpression and overall survival (OS) of lung cancer stratified by histological type (A), tumor stage (B), detection specimen (C), test method (D) and method to obtain HR (E).** The individual and pooled HR with 95% CI was shown by forest plot. HR > 1 implied worse OS for the group with high IL-17 expression.

The results of the meta-analysis were reported in Table 3 and in Figs (2–6). Overall, six studies including 479 patients reported data on IL-17 expression and OS in LC. The combined analysis of the six studies showed that expression of IL-17 was significantly correlated with OS in LC (HR = 1.82, 95% CI: 1.44, 2.29; P < 0.00001). Furthermore, there was no heterogeneity among these studies (I^2^ = 30%, P = 0.21) (Fig 2), and a publication bias became absent when visually identifying the funnel plot (Fig 3). As for DFS, the HR was 2.41 (95% CI: 1.42, 4.08; P = 0.001; I^2^ = 0%) (Fig 4) and a publication bias was not obvious (Fig 5). The fixed-effects model was used because of no significant heterogeneity was observed across these studies. This suggests that IL-17 positive expression may be a significant predictor of poor prognosis for LC patients.

**Table 3:** A summary of hazard ratio (HR) for the overall and subgroup analyses of IL-17 expression and overall survival (OS) of lung cancer patients.

						Heterogeneity	
Subgroup	Studies (n)		Patients(n)	Pooled HR (95% CI)	P -value	P -value	I^2^ (%)
**OS**							
**Overall**	6		479	1.82 [1.44, 2.29]	P < 0.00001	P = 0.21	I^2^ = 30%
**Histological type**							
NSCLC	4		325	1.61 [1.25, 2.09]	P = 0.003	P = 0.42	I^2^ = 0%
SCLC	1		76	3.06 [1.49, 6.27]	P = 0.002		
NSCLC and SCLC	1		78	3.04 [1.35, 6.86]	P = 0.007		
**Tumor stage**							
Ⅰ-Ⅲ	2		154	1.48 [1.10, 1.99]	P = 0.009	P = 0.25	^2^
Ⅰ-Ⅳ	3		247	2.43 [1.58, 3.73]	P < 0.00001	P = 0.71	I^2^ = 0%
Ⅳ(M1a/M1b)	1		78	3.04 [1.35, 6.86]	P = 0.007		
**Detection specimen**							
Intratumoral tissue	2		154	1.48 [1.10, 1.99]	P = 0.009	P = 0.25	I^2^ = 24%
Serum	3		247	2.43 [1.58, 3.73]	P < 0.00001	P = 0.71	I^2^ = 0%
Pleural effusion	1		78	3.04 [1.35, 6.86]	P = 0.007		
**Detection method**							
IHC	2		154	1.48 [1.10, 1.99]	P = 0.009	P = 0.25	I^2^ = 24%
ELISA	4		325	2.55 [1.75, 3.73]	P < 0.00001	P = 0.82	I^2^ = 0%
**Method to obtain HR**							
Reported	5		377	2.51 [1.78, 3.55]	P < 0.00001	P = 0.92	I^2^ = 0%
Estimated	1		102	1.39 [1.01, 1.90]	P = 0.04		
**DFS**	2		95	2.41 [1.42, 4.08]	P = 0.001	P = 0.94	I^2^ = 0%

Abbreviation: NSCLC: non-small cell lung cancer; SCLC: small cell lung cancer; IHC: immunological histological chemistry; ELISA: enzyme linked immunosorbent assay; HR: hazard ratio; OS: overall survival; DFS: disease free survival; HR: hazard ratio; CI: confidence interval.

In subgroup analysis, we analyzed the studies by histological type, disease stage, detection specimen, test method and method to obtain HR reported in Fig 6. When we limited the first subgroup analysis factor, the meta- analysis showed that high IL-17 expression had a poor survival in NSCLC with combined HR 1.61 (95% CI: 1.25, 2.09). Next, we also observed a statistically significant influence of IL-17 expression on poor prognosis in stage Ⅰ-Ⅲ patients with an HR of 1.48 (95% CI: 1.10, 1.99), in stage Ⅰ-Ⅳ patients with an HR of 2.43 (95% CI: 1.58, 3.73) and Ⅳ(M1a/M1b) with 3.04 (95% CI: 1.35, 6.86). Furthermore, subgroup analysis stratified according to detection specimens revealed that the combined HR was 2.43 (95% CI: 1.58, 3.73) in studies with serum samples and in studies with intratumoral tissues there was similar statistical significance with combined HR 1.48 (95% CI: 1.10, 1.99). Similarly, we aggregated the researches separately according to the method used to detect IL-17 expression. The pooled HR was 2.55 (95% CI: 1.75, 3.73) with ELISA and 1.48 (95% CI: 1.10, 1.99) with IHC, both methods were statistically significant. Finally, further stratification by the different methods to obtain HR revealed that the significant association existed with pooled HR 2.51 (95% CI: 1.78, 3.55) by acquired HR directly from the article. The heterogeneity of every subgroup meta-analysis was no significant and we used the fixed effect model to evaluate them.

### Sensitivity analysis

Sensitivity analysis was conducted to examine the reliability of our conclusion. We removed one study at a time and the results were not significantly changed (Table 4). This shows that the conclusion of our meta-analysis is reliable on OS.

**Table 4:** Sensitivity analysis results on overall survival (OS).

		Heterogeneity	
Excluded study	HR (95% CI)	P value	I^2^ (%)
**None**	1.82 [1.44, 2.29]	P = 0.21	I^2^ = 30%
**Chen X [19]**	1.78 [1.40, 2.26]	P = 0.15	I^2^ = 41%
**Zhang GQ[20]**	2.51 [1.78, 3.55]	P = 0.92	I^2^ = 0%
**Xu CH[21]**	1.78 [1.40, 2.26]	P = 0.15	I^2^ = 41%
**Xu CH[22]**	1.74 [1.36, 2.21]	P = 0.24	I^2^ = 27%
**Pan B[23]**	1.79 [1.40, 2.29]	P = 0.13	I^2^ = 43%
**Lin QY[24]**	1.71 [1.34, 2.18]	P = 0.29	I^2^ = 19%

Abbreviation: HR: hazard ratio; CI: confidence interval.

### Publication bias

The funnel plot was performed for detecting publication bias in the meta-analysis. In all included studies, no funnel plot asymmetry was found. These results suggest that there is no publication bias at work.

## Discussion

Chronic inflammation is a crucial factor in the pathogenesis of many different types of cancer, including LC [29]. IL-17 is a pro-inflammatory cytokine which is primarily produced by activated CD4(+) T helper cells known as Th17 cells [30]. There were accumulating results showing that pro-inflammatory cytokines play an important anti-tumorigenic role, but other evidence indicated that these cytokines contributed to the growth and spread of human malignancies [31]. Recently, increasing evidence has suggested that IL-17 expression was elevated in multiple cancer types, including prostate cancer [32], colorectal cancer [33], hepatocellular carcinoma [34], breast cancer [35], ovarian cancer [36] and NSCLC [37]. IL-17 can promote tumor progression via an increase in angiogenesis [38] and prevent cancer cells from immune surveillance [39]. However, IL-17 is correlated with various anti-tumor mechanisms. It can reduce tumor growth and metastasis by suppressing tumor progression via enhanced antitumor immunity [40,41]. Currently, there were several studies evaluating the prognostic value of IL-17 in LC, but none of them came to a conclusive result. We investigated these studies and conducted a meta-analysis in order to evaluate the correlation between IL-17 and prognosis in LC patients.

To the best of our knowledge, this research conducted meta-analysis on assessing the prognostic significance of IL-17 expression in LC for the first time. The results of the present meta-analysis revealed that high expression of IL-17 were significantly correlated with a shorter OS and poorer DFS in LC patients, suggesting that increasing expression of IL-17 may be considered as a significant biomarker in predicting the poor clinical outcome of LC. In subgroup by histological type (NSCLC and SCLC), we discovered a statistically significant unfavorable impact of IL-17 on prognosis within both of them. Furthermore, when the subgroup analyses were performed according to histological type, tumor stage, detection specimen, test method and method to obtain HR, all the results were significant.

The current systematic review of the literatures with meta-analysis indicated that IL-17 is a poor prognostic indicator for survival in LC patients. Although two meta-analyses had been published in 2014 [42] and 2015 [43], some results in their work still need to be advanced: Firstly, this two analyses only examined the correlation between IL-17 expression and clinical outcome in NSCLC patients. Secondly, the number of included researches in their analyses were small (both 3). Thirdly, it was not reported that high IL-17 level was relevant with a poor survival outcome for DFS in NSCLC patients. Hence, it is necessary to conduct a more accurate systematic analysis to demonstrate the prognostic value of IL-17 in LC. Our study has updated that previous meta-analysis by including more recent relevant articles and by generally using a more comprehensive search strategy. Screening and study selection were executed independently and reproducibly by two researchers. In addition, our study evaluates the combined HR for subgroups divided according to histological type, tumor stage, detection specimen, test method and HR estimated method. We also explored heterogeneity and potential publication bias in accordance with published guidelines.

When subgroup analysis was defined according to tumor stage, we found a statistically significant impact of IL-17 on unfavorable survival, revealing that this poor prognostic factor could be of importance not only in early-stage LC but also in advanced staged LC. Furthermore, we observed that the combined HR (3.04) was higher in stage Ⅳ patients than the combined HR (1.48) in stage Ⅰ-Ⅲ patients and the one (2.43) in stage Ⅰ-Ⅳ patients, suggesting that IL-17 expression could be an important adverse prognostic factor for advanced-stage LC.

When we performed subgroup analysis according to detection specimen (intratumoral tissue, serum or pleural effusion), we did not find a significant difference. Moreover, the method used to detect the expression of IL-17 did not have an effect on significance; results were similar for studies that used ELISA or IHC. Those suggest that using samples of peripheral blood or pleural effusion to detect the expression of IL-17 is considered much more cost-effective and convenient in clinical work because body fluid are much easier to acquire from patients than those of tumor tissues. In addition, the peripheral IL-17 can be more easily measured before surgery and provide survival prediction for patients with unresectable tumors.

Sensitivity analysis demonstrated that the correlation between the high expression of IL-17 and LC survival outcome was stable and unchanged after removing any one article. Overall, the results of the present meta-analysis show that elevated expression of IL-17 exerts a significantly negative influence on the prognosis for LC patients. Although, future investigations and validation are needed, these data may provide a new insight into the biological significance of IL-17 in China patients with LC.

Publication bias is a well known problem for all forms of meta-analysis. Although the current analysis did not appear publication bias, other potential biases could not be entirely eliminated. Since our analysis was restricted to the fully published articles, we tended to minimize publication bias by conducting the literature search as comprehensive as possible. However, we did not take unpublished papers, comment and abstracts into account because the required resource was unavailable, which may also lead to publication bias. Besides, positive results are inclined to be accepted by journals while negative results are often rejected or not even submitted. Nevertheless, no significant publication bias was observed, revealing that the statistics obtained approximate the actual results.

To appreciate our study better, several limitations should be considered. First, the test methods were different among these articles. Four studies used ELISA to detect the level of IL-17 in body fluid. Two studies performed IHC to test IL-17 positive cells. Although these studies were all relevant to IL-17 expression, they might lead to clinical and statistical heterogeneity and the sensitivity of those methods is changed. Furthermore, in the articles, cut-off values were also different, which might therefore interpret the inconsistencies observed. Second, all studies containing in this meta-analysis come from one country, the representation is low. This distribution pattern of the populations certainly cannot mean the real situation. Therefore, the conclusions need to be further verified in more racial groups with large sample size researchers. Third, this analysis included only one study concerning SCLC, and the data were insufficient to evaluate the prognostic value in SCLC. Thus, to be sure, further researches were warranted to complete the information. Finally, some data for HR were extrapolated from other available data or survival curves rather than provided directly. Although the method of extracting HR and 95% CI is widely accepted, it might be not completely consistent with the real values. We still need further studies to analyze the role of IL-17 as a prognostic factor for survival in LC.

## Conclusions

In conclusion, despite the limitations of the current study, our systematic review and meta-analysis suggests that high IL-17 expression is an accelerating factor in the development of LC and is correlated with a poor prognosis in Chinese patients with LC. IL-17 could be developed as a novel biological marker contributing to the diagnosis and prognostic evaluation in the LC patients, as well as a potential therapeutic target for LC [44].

So the detection of IL-17 expression may be helpful for us to identify patients from high risk and to develop specific treatment for them. To become an useful prognostic marker for clinical application, the further investigation involving large population-based multicenter prospective studies are required to clearly verify and expand on our conclusion.

## Supporting information

S1 File PRISMA checklist for the meta-analysis. (DOC)

S2 File PRISMA flow diagram for the meta-analysis. (DOC)

S3 File Search terms and the number of studies identified from the PubMed database. (DOC)
