# Supplementary material for: The prognostic value of interleukin-17 in lung cancer: A systematic review with meta-analysis based on Chinese patients
Source: PLoS One. 2017 Sep 21;12(9):e0185168. doi: 10.1371/journal.pone.0185168 (PMC5608354; doi:10.1371/journal.pone.0185168)
Supplement: S2 File — (DOC) [file pone.0185168.s002.doc]

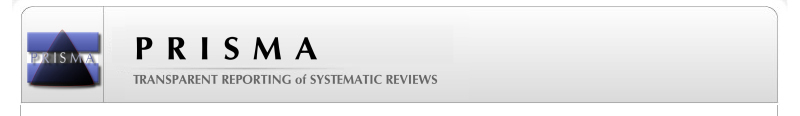
**PRISMA 2009 Flow Diagram**

**Screening**

**Included**

**Eligibility**

**Identification**

Records identified through database searching
(n = 189 )

Additional records identified through other sources
(n = 0 )

Records after duplicates removed
(n =44)

Records screened
(n = 145 )

Records excluded
(n = 113 )

Full-text articles assessed for eligibility
(n = 32 )

Full-text articles excluded, with reasons
(n =26)

Studies included in qualitative synthesis
(n = 6 )

Studies included in quantitative synthesis (meta-analysis)
(n = 6 )
