# Supplementary material for: The prognostic value of interleukin-17 in lung cancer: A systematic review with meta-analysis based on Chinese patients
Source: PLoS One. 2017 Sep 21;12(9):e0185168. doi: 10.1371/journal.pone.0185168 (PMC5608354; doi:10.1371/journal.pone.0185168)
Supplement: S3 File — (DOC) [file pone.0185168.s003.doc]

**S3 Table. Search terms and the number of studies identified from the PubMed Database**.

| **Query** | **Results** |
| --- | --- |
| #1 "Lung Neoplasms"[Mesh] | 200,855 |
| #2 (((Lung tumor[Title/Abstract] OR Lung cancer[Title/Abstract]) OR Lung neoplasm[Title/Abstract]) OR Lung carcinoma[Title/Abstract]) OR Lung oncology[Title/Abstract] | 135,876 |
| #3 (((Pulmonary tumor[Title/Abstract] OR Pulmonary cancer[Title/Abstract]) OR Pulmonary neoplasm[Title/Abstract]) OR Pulmonary carcinoma[Title/Abstract]) OR Pulmonary oncology[Title/Abstract] | 3,278 |
| #4 #1 OR #2 OR #3 | 241,737 |
| #5 "Interleukin-17"[Mesh] | 8,316 |
| #6 (Interleukin-17[Title/Abstract] OR IL-17[Title/Abstract]) OR IL17[Title/Abstract] | 13,039 |
| #7 #5 OR #6 | 15,388 |
| #8 "Prognosis"[Mesh] | 1,343,379 |
| #9 "Survival" [Mesh] | 4,386 |
| #10 "Mortality"[Mesh] | 328,153 |
| #11 ((Prognosis[Title/Abstract] OR Survival[Title/Abstract]) OR Mortality[Title/Abstract]) OR Outcome[Title/Abstract] | 2,056,722 |
| #12 #8 OR #9 OR #10 OR #11 | 2,934,614 |
| #13 #4 AND #7 AND #12 | 42 |
| #14 #13 AND ("0001/01/01"[PDAT] : "2017/04/01"[PDAT]) | 40 |

Final search strategy: ((("Lung Neoplasms"[Mesh] OR ((((Lung tumor[Title/Abstract] OR Lung cancer[Title/Abstract]) OR Lung neoplasm[Title/Abstract]) OR Lung carcinoma[Title/Abstract]) OR Lung oncology[Title/Abstract])) OR ((((Pulmonary tumor[Title/Abstract] OR Pulmonary cancer[Title/Abstract]) OR Pulmonary neoplasm[Title/Abstract]) OR Pulmonary carcinoma[Title/Abstract]) OR Pulmonary oncology[Title/Abstract])) AND ("Interleukin-17"[Mesh] OR ((interleukin-17[Title/Abstract] OR IL-17[Title/Abstract]) OR IL17[Title/Abstract]))) AND ((("Prognosis"[Mesh] OR "Survival"[Mesh]) OR "Mortality"[Mesh]) OR (((Prognosis[Title/Abstract] OR Survival[Title/Abstract]) OR Mortality[Title/Abstract]) OR Outcome[Title/Abstract])) AND ("0001/01/01"[PDAT] : "2017/04/01"[PDAT])
